# In Vitro Investigations in a Biomimetic Approach to Restore One-Piece Zirconia Implants

**DOI:** 10.3390/ma14164361

**Published:** 2021-08-04

**Authors:** Reto Nueesch, Sabrina Märtin, Nadja Rohr, Jens Fischer

**Affiliations:** Biomaterials and Technology, Department of Reconstructive Dentistry, University Center for Dental Medicine Basel UZB, University of Basel, 4058 Basel, Switzerland; nueesch.reto@unibas.ch (R.N.); sabrina.maertin@uzb.ch (S.M.); nadja.rohr@unibas.ch (N.R.)

**Keywords:** mesostructure, suprastructure, polymer-infiltrated ceramic, feldspathic ceramic, cementation, ceramic implant

## Abstract

The objective of this study was to evaluate the fracture load and retention force of different bonding systems while restoring one-piece zirconia implants with a novel cementation approach using a mesostructure. Polymer-infiltrated ceramic mesostructures (*n* = 112) were therefore designed as caps on the implant abutment, and a molar feldspathic ceramic crown was constructed on top of it as a suprastructure. For cementation, different bonding systems were used. Fracture load and retention force were measured immediately after storage in water at 37 °C for 24 h (*n* = 8) as well as after artificial aging in a chewing simulator and subsequent thermal cycling (*n* = 8). Combined restorations showed higher fracture load compared to monolithic restorations of polymer-infiltrated ceramic (*n* = 8) or feldspathic ceramic (*n* = 8) identical in shape. However, the fracture load of the combined restorations was significantly affected by aging, independent of the primers and cements used. Restorations cemented with primers containing methyl methacrylate and 10-methacryloyloxydecyl dihydrogen phosphate exhibited the highest retention force values. Aging did not affect the retention force significantly. Similar fracture load values can be expected from combination restorations when compared with monolithic crowns.

## 1. Introduction

Due to successful osseointegration as well as clinical reliability and based on well-documented scientific data, commercially pure titanium or its alloys with a moderately rough surface are the gold standard for dental implants [1].

With a survival rate of 94.3–98.4% after 3–5 years of functional loading and a mean peri-implant bone loss of 0.7–1.0 mm [2,3,4], implants made from zirconia (ZrO_2_) have been introduced as a possible alternative to titanium implants [5,6,7,8,9,10].

Today, cement-retained ceramic crowns are the common restorations used on one-piece zirconia implants. Clinical studies have reported unacceptable rates of veneer chipping of up to 47% after up to five years for one-piece zirconia implant-supported veneered zirconia crowns [8,11,12,13]. Therefore, veneered zirconia is not the material of choice to restore ceramic implants.

In contrast, clinical results for zirconia implant-supported monolithic lithium disilicate restorations showed a survival rate of 100% after five years. In 2 out of 22 restorations, major occlusal roughness was observed; thus, the authors report a success rate of 90.9% [14]. Laboratory results testing monolithic solutions for the restoration of one-piece zirconia implants with zirconia, lithium disilicate, and even more elastic materials such as polymer, composite, or polymer-infiltrated ceramic suggest that all tested monolithic materials can be recommended for use on zirconia implants from a mechanical point of view [15,16,17,18,19,20], provided that a resin composite cement with high compressive strength is used for cementing the restoration on the abutment [16,17,20].

As margins of one-piece zirconia implant-supported crowns are placed subgingivally, the removal of excess cement is challenging. Undetected cement quantity increases when the restoration margins are located more subgingivally [21,22]. Cement residues in the soft tissue are suspected of inducing periimplantitis [23,24,25]. A second surgical intervention might be performed after cementation to remove the cement residues; however, this procedure is invasive. Crown venting is the most effective technique to avoid cement excess; however, currently, there is no technique available to avoid cement excess [26] entirely.

The basic idea to eliminate excess cement with one-piece implants is to cement a mesostructure on the abutment after implant placement and prior to wound closure, allowing to remove the excess cement under direct visual control. After the healing process, the mesostructure is intraorally prepared and restored with a crown as a suprastructure, this process being similar to the restoration of a natural tooth. Laboratory investigations suggest that the fracture load of crowns fabricated from polymer-infiltrated ceramic is higher than the masticatory forces [18]. Besides its strength, this material offers a high damage tolerance [27], which is advantageous in regard to the milling process. Therefore, polymer-infiltrated ceramic was selected as the material for the mesostructure. For the suprastructure, feldspathic ceramic was chosen because it provides sufficient strength and is easy to grind, requiring no further production step except polishing.

To bond the three different materials—zirconia, polymer-infiltrated ceramic, and feldspathic ceramic—appropriate chemistry of the adhesives is required. It is known that 10-methacryloyloxydecyl dihydrogen phosphate (MDP) provides high bond strength to zirconia [28,29]. On the other hand, a silane is required on the silicate-based feldspathic ceramic as well as on the ceramic part of the polymer-infiltrated ceramic [28]. And finally, on the resin portion of the polymer-infiltrated ceramic, a stable bond can be achieved with methyl methacrylate [30]. Therefore, these three chemical compounds were used for the tests.

As the polymer-infiltrated ceramic exhibits a modulus of elasticity of 35.5 GPa [27], which corresponds to dentin (12–45 GPa) [31], and the feldspathic ceramic has a modulus of elasticity of 72.1 GPa [27], which corresponds to enamel (40–130 GPa) [31], this material combination may be classified as a biomimetic approach to restoring one-piece zirconia implants.

The aim of the present study was (1) to test a potential solution for an excess-free cementation process on one-piece zirconia implants in regard to fracture load and retention force and (2) to test different bonding systems in this application.

## 2. Materials and Methods

### 2.1. Implant Preparation

One hundred and twenty-eight one-piece zirconia implants (ceramic.implant, VITA, Bad Säckingen, Germany) with a diameter of 4.0 mm and a length of 8.0 mm in the intraosseous part were used. The implant abutments had a machined surface exhibiting a roughness of R_a_ = 0.42 ± 0.06 μm (*n* = 10; T8000, Hommel Wave, Schwenningen, Germany). All implants were embedded in epoxy resin (RenCast CW 20/Ren HY 49, Huntsman Advanced Materials, Duxford, UK) according to ISO 14801:2016. Following the standard, there was a 3 mm clearance between the implant neck and the epoxy surface to simulate alveolar bone resorption. Specimens not subjected to aging were fixed in a cube-shaped silicon mold (internal dimensions: width: 35 mm, length: 35 mm, height: 35 mm). Specimens subjected to aging procedures were embedded in a special truncated cone-shaped silicon mold (internal dimensions: upper diameter: 20 mm, lower diameter: 16 mm, height: 35 mm) to be mounted in the holder of the chewing simulator. After embedding, all specimens were stored in a drying oven (Memmert UE200, Memmert, Schwabach, Germany) at 60 °C for 14 h.

### 2.2. Production of the Restorations

**Figure 1:**
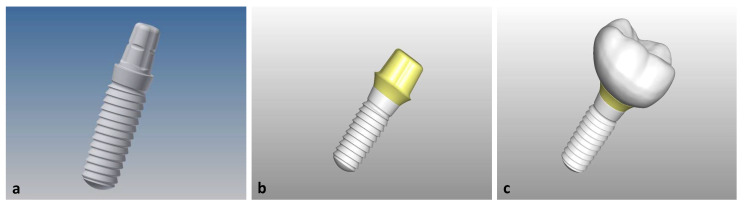
(**a**) Implant after manual entry of the geometries in the software Inventor; (**b**) imported mesostructure in the Freeform software on the before constructed implant; (**c**) Representation of the suprastructure constructed on the mesostructure.

Based on the technical drawing of the implant, its geometry was transferred to a 3D-design system (Inventor Professional 2019, Autodesk, San Rafael, CA, USA), and a mesostructure was designed on the abutment with a wall thickness of 1 mm and a shoulder to hold a crown. The design of the mesostructure was imported into the 3D-software Freeform (Geomagic Freeform Plus, 3D-Systems, South Carolina, USA), and a molar crown (tooth 46) was constructed on top of it as suprastructure (Figure 1).

By means of high-precision machining (Ultrasonic 20 linear, DMG MORI, Tokyo, Japan) 112 mesostructures identical in shape were pre-milled and ground subsequently with three different tools (VHM-Torusfräser, ZECHA Hartmetall Werkzeugfabrikation, Königsbach-Stein, Germany; 120_US-D2-D126-Kugelschleifer, and 143_D1-D76- Kugelschleifer, Emuge-Werk Richard Glimpel, Lauf an der Pegnitz, Germany) from a polymer-infiltrated ceramic (VITA Enamic, VITA).

One hundred and twelve suprastructures were manufactured from feldspathic ceramic (Vitablocs Mark II, VITA) with three different ball-grinders (120_US-D2-D126, 121_US-D3-D126, and 143_D1-D76, Emuge-Werk Richard Glimpel). The occlusal surface was polished with a rubber-polisher (Twist Polisher, Hager & Meisinger, Neuss, Germany), a goat-hair brush, and a diamond polishing paste (Karat Diamond Polishing Paste, VITA).

A monolithic full-contour restoration was designed by merging the meso- and suprastructure without geometric changes (Power Shape, Autodesk). Eight monolithic restorations of polymer-infiltrated ceramic and feldspathic ceramic each were fabricated analog to the mesostructures (polymer-infiltrated ceramic) and suprastructures (feldspathic ceramic).

### 2.3. Cementation of the Restorations

Prior to cementation, all meso- and suprastructures, as well as the monolithic restorations, were cleaned in an ultrasonic bath (Transsonic T310, Elma Schmidbauer, Singen, Germany) with ethanol for 60 s. The abutment portions of the implants were cleaned with a foam pellet soaked in ethanol. Table 1 details the main compositions of the primers and cements as provided by the manufacturers.

**Figure 2:**
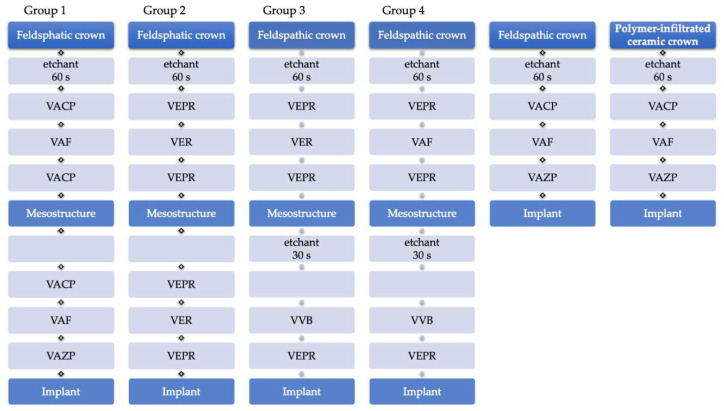
Test matrix describing the cementation procedure of mesostructures on implants and feldspathic crown on mesostructures (Group 1–4) and the cementation of monolithic feldspathic crowns and polymer infiltrated network crowns on implants. *n* = 16 per test-group; 8 initial, 8 aged.

The intaglio surfaces of the suprastructures and monolithic restorations were acid-etched for 60 s with hydrofluoric acid (VITA Adiva Etch, VITA), cleaned with oil-free water-spray, ultrasonically rinsed in 40 mL distilled water (Transsonic T310, Elma Schmidbauer, Singen, Germany), and dried with oil-free air. All specimens were produced successively, one after the other. Pre-treatment of abutments and meso- and suprastructures, as well as cementation of the monolithic restorations, was performed according to the test matrix presented in Figure 2, following the manufacturers’ instructions by the same operator (SM). When an adhesive composite was used, the first 0.5 cm of the material was discarded.

After applying the respective primer and cement, mesostructures were placed on the abutment with finger pressure. Excess cement was removed with foam pellets. Suprastructures were cemented on the mesostructures using the same approach. The procedure for cementing the monolithic restorations was alike. The specimens were placed in an alignment apparatus at room temperature, and a force of 20 N was applied for 15 min. After cementation, the specimens were stored in deionized water at 37 °C for 24 h (WTC binder, Binder, Tuttlingen, Germany).

### 2.4. Artificial Aging

After storage in water for 24 h, 16 specimens of Groups 1, 2, and 4, as well as eight specimens of Group 3 were loaded in sets of eight in a chewing simulator (Chewing Simulator CS-4, SD-Mechatronic, Feldkirchen-Westerham, Germany) parallel to the implant axis in the central fissure with a steel ball (diameter: 4.5 mm) with 1.2 million cycles, a load of 49 N, and a frequency of 1.5 Hz.

Subsequently, the specimens underwent thermal cycling for 10,000 cycles between 5 and 55 °C with a 25 s dwell time and a 5 s transfer time. To avoid buoyancy in the thermocycler (Thermocycler THE 1200, SD-Mechatronics), the specimens were stored in perforated plastic tubes, which were screwed to the bottom of the basket.

### 2.5. Testing

Retention force of Groups 1, 2, and 4, and fracture load of Groups 1–4 were measured immediately after water storage (“initial”) or thermal cycling (“aged”). Monolithic restorations were only measured for fracture load immediately after water storage; no aging was performed. A sample size of *n* = 8 per group was chosen according to the results of previous studies using a similar test set-up [2,18,20].

#### 2.5.1. Retention Force

**Figure 3:**
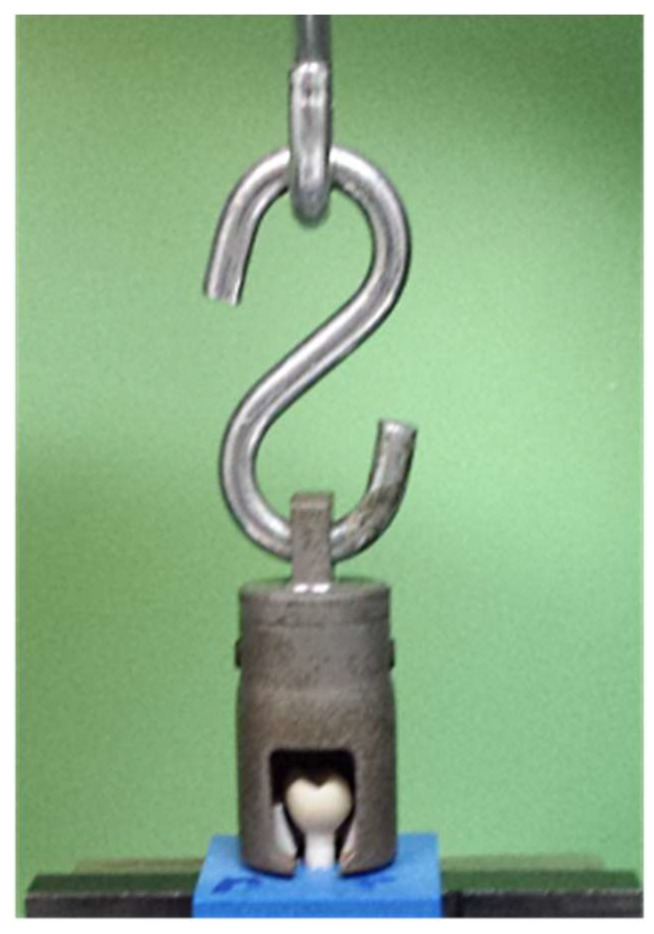
Trigger bell for the measurement of retention force, lined with a molded Teflon inlay.

To measure the retention of the restorations on the abutments, all specimens were successively placed in the universal testing device (Z020, Zwick/Roell, Ulm, Germany) in a customized specimen mount, which was lined with a molded Teflon inlay to avoid force peaks (Figure 3). A tensile force was applied with a crosshead speed of 1 mm/min, and force at debonding of the restoration was recorded. The implants were inspected for cement residues with a light microscope (Stereomikroskop Mantis Compact Universal, Vision Engineering, Emmering, Germany) at a magnification of 8×.

#### 2.5.2. Fracture Load

To measure the fracture load, specimens were loaded parallel to the implant axis in the central fissure with a steel ball (diameter: 4.76 mm). To avoid force peaks, a 0.2 mm thick tin foil (Dentaurum, Pforzheim, Germany) was placed between the steel ball and the occlusal surface of the restoration. All specimens were successively placed in the universal testing device (Z020), and force was applied until fracture with a crosshead speed of 1 mm/min. Load at fracture was recorded. After fracture, the implants were inspected for cement residues with a light microscope (Stereomikroskop Mantis Compact Universal) at a magnification of 8×.

### 2.6. Compressive Strength of the Cement Materials

The compressive strength of the two cements used was measured with cylindrical test specimens (3.0 mm height, 3.2 mm diameter, *n* = 12). Cement was filled into the respective cavities of a Teflon mold (Distrelec, Nänikon, Switzerland), covered with a plastic foil and a glass plate on each side, and kept in a dimmed room for 10 min. The molds were placed in distilled water at 37 °C for 1 h. The specimens were carefully removed from the mold, controlled with a digital caliper (M823-160, Brüder Mannesmann, Remscheid, Germany), and stored for a further 23 h in the water bath at 37 °C. Twelve specimens of each cement were produced and loaded parallel to the cylinder axis until fracture (Z020) with a crosshead speed of 1 mm/min.

Compressive strength was calculated with the equation:

$$\sigma_{c}=\frac{F}{A}$$

*σ_c_*: compressive strength; *F*: fracture load; *A*: cross section = πr^2^ (*r*: radius = 1.6 mm).

### 2.7. Statistical Analysis

Two-way ANOVA was performed to test for differences between groups and the effect of aging, followed by a post-hoc Fisher LSD test (Stat Plus Pro V.6.1.25, Analyst Soft, Walnut, CA, USA). The level of significance was set at α = 0.05. Results are shown as mean ± standard deviation.

## 3. Results

No damage was detected on any specimen after artificial aging. For the evaluation, data of all 128 specimens, therefore, could be applied. All *p*-values are listed in Appendix A (Table A1 and Table A2).

### 3.1. Retention Force

A significant effect was found between groups (*p* < 0.01), but not for aging (*p* = 2.26). Specimens of Groups 1, 2, and 4 were tested. In Groups 1 and 2, all restorations detached completely from the abutment. No cement residues could be detected on the implant abutment for these groups; in Group 4, fragments of the restoration adhered to the abutment. Failures between meso- and suprastructures were not observed in any group.

**Figure 4:**
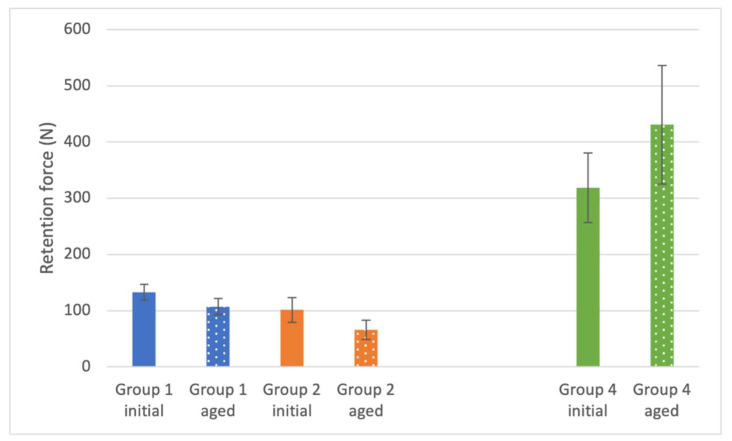
Retention force of the restorations (*n* = 8 per group).

Initial retention forces of Group 1 (132.9 ± 14.1 N) and Group 2 (101.4 ± 22.2 N) did not differ significantly from each other. However, a significant difference was found for both groups when compared to the result of Group 4 (319.0 ± 61.8 N) (Table 2 and Table A1, Figure 4).

After artificial aging, a significant reduction in retention force was observed for Group 1 (107.3 ± 14.2 N) and Group 2 (65.7 ± 17.1). The retention force of Group 4 (430.9 ± 105.6 N), however, significantly increased after aging (Table 2 and Table A1, Figure 4).

### 3.2. Fracture Load

A significant effect was found between groups (*p* < 0.01), and for aging (*p* < 0.01). The fracture load test resulted in the total destruction of all restorations with 2–4 fragments. In Groups 1 and 2, no cement residues were detected on the abutment, whereas in Groups 3 and 4, fragments or the whole fractured restoration adhered to the implant.

**Figure 5:**
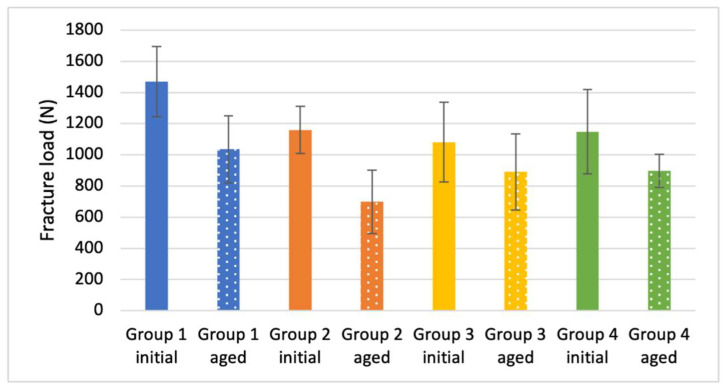
Fracture load values of the restorations (*n* = 8 per group).

The initial fracture load values for Group 1 (1469.8 ± 224.5 N) were significantly higher than those for Group 2 (1159.6 ± 151.1 N), Group 3 (1081.8 ± 256.0 N), and Group 4 (1148.3 ± 269.5 N), which differed not significantly among each other (Table 3 and Table A2, Figure 5).

Artificial aging resulted in a decrease of fracture load, which was significant for Groups 1, 2, and 4.

The fracture load values of the monolithic restorations differed significantly from each other (feldspathic ceramic: 951.4 ± 172.2 N; polymer-infiltrated ceramic: 1186.6 ± 247.7 N) (Table 3 and Table A2, Figure 5). The values for both monolithic restorations were significantly lower when compared to the initial measurements of Group 1 but not different from the values of Group 2–4 (Table 3 and Table A2, Figure 5).

### 3.3. Compressive Strength of Cements

The compressive strengths of the two cements are listed in Table 4. The value of VAF was higher by 13.4%; however, there was no significant difference compared to VER (*p* = 0.061).

It was not possible to produce homogeneous specimens of VVB in the required dimensions to measure its compressive strength because the material has a low viscosity and bubbles formed during polymerization in the bulk. Therefore, no compressive strength testing was performed for VVB.

## 4. Discussion

The aim of the present study was to test a concept for an excess-free cementation process on one-piece zirconia implants in vitro. The advocated concept is based on a restoration consisting of a meso- and a suprastructure. The solution was examined in regard to retention force and fracture load. Further, an experimental resin composite cement and the corresponding primer, as well as a methyl methacrylate containing bonder, were tested in this specific application.

Suprastructures and monolithic restorations were designed by a CAD/CAM-technician; thus, the shaping may be regarded as equivalent to restorations used clinically in dental practice. Due to the standardized laboratory approach, prefabricated mesostructures were used with an identical outer aspect and a uniform circular shoulder. To achieve long-lasting bonding with high bond strength to zirconia, air-particle abrasion of the zirconia surface prior to application of the primer is recommended, thus increasing the surface area and inducing higher surface energy [32]. However, intraoral air-particle abrasion may cause damage to the surrounding tissues and therefore is not feasible. Consequently, in this in vitro study, the abutment of the zirconia implant was pretreated with a primer only.

An effect of artificial aging was observed. However, it did not reach significance in every case. Probably the chewing simulation, with a load parallel to the axis of the implant but without lateral movement, does not sufficiently mimic the clinical situation. Further, the shape of the restoration is individual and makes it difficult to compare the results with other studies. Nevertheless, the results provide a base to compare different material combinations and may be regarded as a valuable in vitro test method.

The retention force of monolithic restorations was not measured in the present study, but the results of a previous study [18] may be used for comparison. The cementation process to fix the restoration on the abutment was the same in both studies, and after testing the cement layer in both studies, stuck to the intaglio surface of the restoration, implying that the failure modes are identical and results may be compared. The initial retention force of Group 1 (132.9 N) was less than the retention force of the monolithic polymer-infiltrated ceramic restorations in the previous study, where a mean retention force of 159 N was reported. In the previous study, 4.5 mm diameter implants were used, which provide a greater abutment surface, thus explaining the slightly higher values in that investigation. However, after aging, the retention force in the present study decreased by 19.3%, whereas in the previous study, no decrease was observed. No damage was detected in the bond between meso- and suprastructure. Therefore, the decrease in retention force after aging observed in the present study might be due to the difference in the elastic moduli of both restorative materials as this is—apart from the different implant diameters—the only difference between both test designs.

Initial values in Group 2 were slightly but not significantly lower than those of Group 1. The slightly lower retention force of Group 2 compared to Group 1 might be attributed to the application of a universal experimental primer (VEPR), which was tested in purpose to facilitate the procedure for the practitioner. The retention force of Group 3 was not measured because, in Groups 3 and 4, bonding between mesostructure and abutment was performed with identical materials. The mesostructure of Group 4 was etched to increase the resin surface because VVB, due to its chemistry, does not bond to ceramic. Nonetheless, fragments of the restorations remained on the abutment after the retention test, indicating that the bond strength to zirconia was higher than the bond strength between meso- and suprastucture or the intrinsic strength of the materials themselves. Group 4 showed a 2.4× higher retention force than Group 1. An explanation might be that the MDP molecules probably are much better aligned in the presence of methyl methacrylate, with their hydrophilic phosphate group facing the zirconia surface; the hydrophobic group oriented its direction to the methyl methacrylate layer. After aging, the retention force of Group 4 further increased by 26.0%, which may indicate an ongoing polymerization of the bonding materials during the period of aging.

The fracture load of the monolithic restorations made of polymer-infiltrated and feldspathic ceramics amounted to 1186.6 ± 247.7 N and 951.4 ± 172.2 N, respectively. These values are similar to values measured earlier (1297 ± 150 N and 1042 ± 88 N) using the identical composite resin cement [16]. Hence, it may be concluded that the results obtained with this test method are reproducible.

Mean fracture load values of Group 1 before and after aging are significantly higher than those of Group 2. These findings correspond to the higher compressive strength of the commercially available cement used in Group 1 compared to the experimental cement used in Group 2. The compressive strength has a considerable impact on the fracture load of the restoration [16]. Initial fracture load values of Groups 3 and 4 were similar to that of Group 2. Cementation of the mesostructures in Groups 3 and 4 was performed with VEPR and VVB. For cementation of the suprastructure in Group 3, the experimental resin composite cement VER was used; in Group 4, the commercial product VAF came into play. For Group 1 (cementation with VAF), a significantly higher fracture load was measured than for Group 2 (cementation with VER). No difference was found between Group 3 (cementation of the suprastructure with VAF) and Group 4 (cementation of the suprastructure with VER). It may be concluded that the mechanical properties of the cement between meso- and suprastructure are irrelevant, and only the cement between abutment and mesostructure has an effect on the fracture load. However, the compressive strength of poly-methyl methacrylate is in the range of only 90 MPa [33], the fracture load values of Groups 3 and 4, in contrast, are similar to those of Group 2. It may be hypothesized that the extraordinary high bond strength in Group 4, verified by the retention test, contributes to the high fracture load. But after aging, the bond strength further increased while the fracture load distinctly decreased, which contradicts this hypothesis. Based on the present data, no explanation can be provided for the comparably high fracture load values of Groups 3 and 4.

The initial fracture load value of Group 1 was significantly higher than those of the monolithic restorations. The results indicate that splitting the restoration in meso- and suprastructure does not weaken the restoration in the initial state.

A strong decrease in the fracture load after aging was observed for all Groups 1–4, which is in contrast to our previous study with monolithic polymer-infiltrated ceramic restorations, which were cemented in an identical procedure with identical materials as Group 1 [18]. These earlier results did not exhibit any decrease in fracture load. After testing, fragments in Groups 3 and 4 still adhered to the abutment. Therefore, it may be concluded that the weak link is not the bond between abutment and intaglio surface of the mesostructure but the restorative materials themselves. It may be speculated that the combination of a more elastic mesostructure and a stiffer suprastructure has stressed the materials more than would happen with a monolithic restoration.

In principle, the results suggest that the proposed solution for an excess-free cementation procedure of restorations on a one-piece zirconia implant is a potential approach to avoid cement remnants when restoring one-piece implants. However, the results are not sufficiently conclusive, and further studies have to address the effect of different material combinations in order to learn if the differing moduli of elasticity affect the overall strength of the combined restoration during the aging procedure.

The observation that in Groups 1 and 2, all restorations detached completely from the abutment, while in Groups 3 and 4, the fragments of the restorations remained on the abutment both after the retention test and fracture load test proves that the combination of methyl methacrylate (VVB) and MDP (VEPR) provides extraordinary bond strength, while the cement between meso- and suprastructure has little impact on retention force and fracture load of the complete restoration.

## 5. Conclusions

Within the limitations of the study, it can be concluded that:

- Retention of polymer-infiltrated restorations on zirconia implants may be increased by the application of methyl methacrylate in combination with MDP.
- Retention of polymer-infiltrated restorations on zirconia implants is not affected by aging when methyl methacrylate is used in combination with MDP.
- Fracture load of restorations consisting of a polymer-infiltrated ceramic mesostructure and a feldspathic ceramic suprastructure is significantly reduced by aging, independent of the primers and cements used.
- Further material combinations for meso- and suprastructure have to be investigated prior to a clinical study.

## Figures and Tables

**Table 1:** Composite resin cement and primer used in the study (material compositions according to the manufacturer’s product specification).

Name	Code	Composition
VITA Adiva Etch	etchant	Hydrofluoric acid 5%
VITA Adiva C-Prime	VACP	Silanated methacrylate, Ethanol
VITA Adiva ZR-Prime	VAZP	Adhesive monomers, acetone
VITA Adiva F-Cem	VAF	Mixture of bis-GMA-based resins, Catalysts, Stabilizers, and Pigments
VITA Exp. Primer	VEPR	Experimental primer with MDP
VITA Exp. RCC	VER	Experimental resin composite cement
VITA Vionic Bond	VVB	Methyl methacrylate, 2,2’-ethylenedioxydiethyl dimethacrylate, dibenzoyl peroxide, benzoyl peroxide

**Table 2:** Retention force means and standard deviations. Statistically significant differences between groups determined with Fisher-LSD post-hoc test are indicated with varying superscript letters (*p* > 0.05) (upper case horizontal, lower case vertical).

Groups	Initial (N)	Aged (N)
Group 1	132.9 ± 14.1 ^A,a^	107.3 ± 14.2 ^A,a^
Group 2	101.4 ± 22.2 ^A,a^	65.7 ± 17.1 ^A,a^
Group 3	-	-
Group 4	319.0 ± 61.8 ^A,b^	430.9 ± 105.6 ^B,b^
monolithic feldspathic restoration	-	-
monolithic PICN restoration	-	-

**Table 3:** Fracture load means and standard deviations. Statistically significant differences between groups determined with Fisher-LSD post-hoc test are indicated with varying superscript letters (*p* > 0.05) (upper case horizontal, lower case vertical).

Groups	Initial (N)	Aged (N)
Group 1	1469.8 ± 224.5 ^A,a^	1035.5 ± 216.0 ^B,a^
Group 2	1159.6 ± 151.1 ^A,b,c^	698.4 ± 202.5 ^B,b^
Group 3	1081.8 ± 256.0 ^A,b,c^	890.9 ± 244.6 ^A,a,b^
Group 4	1148.3 ± 269.5 ^A,b,c^	896.7 ± 105.5 ^B,a,b^
monolithic feldspathic restoration	951.4 ± 172.2 ^b^	-
monolithic PICN restoration	1186.6 ± 247.7 ^c^	-

**Table 4:** Compressive strength of the cements.

Material	Compressive Strength (MPa)
VAF	325.1 ± 45.6
VER	286.6 ± 50.1

## Data Availability

The data presented in this study are available on request from the corresponding author.

## Appendix A

**Table A1:** Retention force: *p*-values (colored: significant difference).

Groups	1 Aged	2 Initial	2 Aged	4 Initial	4 Aged
1 initial	0.362	0.263	0.019	<0.001	<0.001
1 aged		0.833	0.142	<0.001	<0.001
2 initial			0.206	<0.001	<0.001
2 aged				<0.001	<0.001
4 inital					<0.001

**Table A2:** Fracture load: *p*-values (colored: significant difference).

Groups	1 Aged	2 Initial	2 Aged	3 Initial	3 Aged	4 Initial	4 Aged
1 initial	<0.001	0.003	<0.001	<0.001	<0.001	0.002	<0.001
1 aged		0.234	0.009	0.655	0.166	0.278	0.183
2 initial			<0.001	0.454	0.011	0.913	0.013
2 aged				<0.001	0.066	<0.001	0.059
3 inital					0.069	0.521	0.077
3 aged						0.015	0.955
4 inital							0.017
